# Management practices and performance of mergers and acquisitions in Pakistan: mediating role of psychological contract

**DOI:** 10.1186/s40064-016-3184-3

**Published:** 2016-09-13

**Authors:** Muhammad Waseem Bari, Meng Fanchen, Muhammad Awais Baloch

**Affiliations:** School of Management and Economics, Beijing Institute of Technology, 5-South Zhongguancun Street, Beijing, 100081 China

**Keywords:** Management practices, Psychological contract, Soft issues, M&A performance, SEM-PLS, Pakistan

## Abstract

The objective of this study is to examine the direct and indirect effect of management practices (procedural justice, coordination approach, communication system, integration strategy, and coping programs) on merger and acquisition (M&A) performance in the Pakistan banking industry. Psychological contract (PC) acts as a mediator between Management practices and M&A performance. The Present study distributes a structured questionnaire to 700 bank employees of different management cadres. The useful response rate is 76 % (536 employees). It uses PLS-SEM technique for data analysis. Findings: (1) procedural justice is a key strategy which has highly significant direct and indirect effect on M&A performance; however integration strategy and the communication system have an only direct effect. (2) PC performs partial mediation at different levels between management practices and M&A financial and non-financial performance. This study provides an effective solution to solve the soft issues during and post-M&A process. This is one of the few studies which effectively integrate the five constructs into a single framework to study their effects on M&A performance. Limitations and future research directions are presented in the last section of the study.

## Background

The aim of every business is to grow, expand, and improve performance. Mergers and acquisitions (henceforth M&A) strategies are performing this role for organizations from last four decades (Bellou 2007). M&A refers to cases of joint activities where minimum two or more, separate legal entities convert into a single entity (Hagedoorn and Duysters 2002; Yan and Zhu 2013). Financial and strategic variables as predictors determine the M&A performance (Giessner et al. 2016; King et al. 2004; Weber and Tarba 2010). The Performance of M&A strategies determines the future of the newborn organization. Scholars describe three dimensions to measure the M&A performance which includes financial (market and accounting performance), non-financial (operational and overall performance) and mixed (Meglio and Risberg 2011). Financial risk, market value, profitability, growth in sales, leverage, and liquidity are most common determinants to measure the M&A financial performance (FP) (Campa and Hernando 2005; Hagedoorn and Duysters 2002; King et al. 2004; Sharma 2013; Smith and Pedace 2011). In contract, market share, innovation, productivity, and attainment of goals are main indicators used to measure the non-financial performance (NFP) of M&A (Brush 1996; Di Guardo et al. 2015; Kapoor and Lim 2007; Musharraf 2003). Research about M&A performance is still patchy and fragmented which indicates gaps waiting to be filled (Weber and Tarba 2010). For instance, numerous studies are conducted under the supposition that M&A are similar in nature, however, in reality, all M&A are not identical (Bower 2001; Joash and Njangiru 2015). A major oversight in this respect has been considered the lack of comparative research on M&A performance in different countries and industries.

In spite of M&A popularity and growth, when the stated goals of the M&A are considered, only 50–60 % M&A are successful (Cartwright and Schoenberg 2006; Weber and Tarba 2010). Soft issues (human resource and culture-related problems) have been widely considered as a prominent reason for post-M&A failure or underperformance (Bauer et al. 2016; Bohlin et al. 2000; Creasy et al. 2009; Dixon 2005). There is a deficiency of such studies which theoretically and empirically investigate the relationship between management practices and M&A performance to handle these soft issues at post integration stage.

The aspects of soft issues are more important in Pakistan where weak corporate culture and feeble judicial system increase the need for better management Practices during and post M&A. The banking sector in Pakistan has been performing a vital role in the financial sector. In 2002, there were total 49 banks working under SBP (State Bank of Pakistan) in different domains (SBP 2003). Now, only 38 banks are working under SBP (SBP 2015). There are several reasons for this significant decline in a total number of banks in Pakistan. For instance, tough competition, high transaction cost, international financial crisis and statutory reforms announced by SBP triggered the small banks to choose the option of M&A. From 2002 to 2011, 57 deals of M&A in the banking industry of Pakistan have completed in which 38 mergers and 19 are acquisition deals (Abbas et al. 2014). Several authors found that M&A performance in Pakistan banking industry is not satisfactory and newborn organizations as a result of M&A could not perform well (Abbas et al. 2014; Haider et al. 2015; Kouser and Saba 2011). For instance, there are two classical case studies of M&A in the context of Pakistan banking sector. In 2007, an acquisition deal between Royal Bank of Scotland (RBS) and ABN AMRO Bank was executed. Internationally, it was one of the biggest deals of acquisition in banking sector and interaction of different cultures (Dutch, British, and US shareholders). The consultants of this deal were much concerned about cultural, employees and unions related problems that may prove this deal difficult, costly, and unfavorable (Santander 2007). In 2011, a research on RBS in the context of Pakistan concluded that after acquisition financial performance and market share of RBS was decreased (Kemal 2011). The results of this case study confirmed the concerns of consultants regarding RBS deal.

The second example is the acquisition of Union bank Ltd. by Standard Chartered Bank Ltd. (Pakistan) in 2006. Union bank was a Pakistan based bank with different organizational culture and employees working style from international culture and operational style of Standard Chartered Bank (Pak) Ltd. These cultural and operational differences between Union Bank and Standard Chartered Bank (Pak) Ltd. effected the post-acquisition performance of Standard Chartered Bank (Pak) Ltd. (Ahmad et al. 2011). In 2012, a study highlighted that Standard Chartered Bank (Pak) Ltd. decreased financial and non-financial performance after the acquisition of Union Bank Ltd. (Arshad 2012). M&A brings transformational changes which create uncertainty, tension and sense of career insecurity among employees (Wan 2015). Ignorance of soft issues during and post-integration stage can be a key reason of M&A failure or under performance (Buiter and Harris 2013; Creasy et al. 2009; Jerjawi 2011); especially in Pakistan where the job market is uneven and banking industry has been squeezed.

In literature, different management practices (e.g. integration strategy, communication system, procedural justice, coordination approach and coping programs) are proposed and used to enhance the M&A performance in different contexts (Ahammad et al. 2016; Basile et al. 2012; Musharraf 2003; Weber and Tarba 2010). For instance, Weber and Tarba, explain that effective communication system has a significant effect on M&A performance in high-tech industry of Israel (Weber and Tarba 2010). Another across countries study concluded that communication system plays a significant role in M&A performance in the context of France, Belgium, Japan and Denmark but against the common wisdom an increase in communication can have negative effects for German acquirers (Weber et al. 2012). A study on Nigerian banking sector concluded that communication system subject to its quality is helpful to enhance the M&A performance and cool down the soft issues (Gomes et al. 2012). Musharraf determined that management practices (coping program, integration plan, procedural justice and communication system) have an effect on M&A performance in the context of Saudi Arabia (Musharraf 2003). Changes in the organization due to M&A may create uncertainty and distrust between employees and employer which may trigger the soft issues and effect on M&A performance. This scenario indicates the need of psychological contract (PC) between employees and employer to make management practices more rewarding and to increase the M&A performance.

The aim of this paper is to investigate the role of management practices in M&A performance and the mediating role of PC to make these management practices more rewarding to the M&A performance in the banking industry of Pakistan. The present paper focuses on the M&A performance in the banking industry of Pakistan, but its findings also have implications in many other developing countries, especially in south Asian.

In view of the high failure rate of M&A, given the lack of comparative research, focus on M&A context and relevant management practices, the present empirical paper focuses on management practices in Pakistan banking industry at post-merger integration stage which may be helpful to solve soft issues, leading to improving M&A performance. The contributions of the present paper are threefold: (1) it proposes management practices to increase the M&A performance; (2) it empirically investigates the specific relationships, if any between proposed management practices in Pakistan banking industry and M&A performance (both, FP and NFP); (3) it investigates the mediating role of PC between management practices and M&A FP and NFP in the context of Pakistan banking industry.

## Theoretical background and hypothesis development

In literature, numerous variables have investigated that predict the M&A performance in different industries. However, results indicate that there is an unclear relationship between financial and strategic variables and M&A performance (King et al. 2004; Yan and Zhu 2013). These findings can be described in many ways. First, employees at the integration stage may prevent to use the latent synergy that can be developed from employees’ interaction and mutual sharing of intangible assets. With reference to social exchange theory and equity theory, employees may avoid sharing their knowledge and skills when they perceive a basic difference in the form of intangible assets (Empson 2001; Weber and Tarba 2010).

Second, post M&A cultural differences between two or more organizations may cause problems such as stress, trauma, and negative behaviors towards the newborn organization and its management (Buiter and Harris 2013; Jerjawi 2011; Weber 1996). Scholars explained that cultural mismatch is the main cause behind poor M&A performance (Cartwright and Cooper 1993, 2014). Various researchers call culture fit as “social glue” which binds individuals and creates organizational harmony (De Silva and Opatha 2015). An analysis of 2700 M&A deals (33 high profile organizations) over three decades determine 50–75 % failure rate of M&A (Kazík 2012) and fix cultural and leadership clashes as the main reasons for M&A failure (Schneider 2008). The negative effects of soft issues on M&A performance in the context of Pakistan banking industry are much stronger because of the high reliance on employees in the service industry, weak corporate culture, lack of management practices and uncertain economic, political and judicial environment. In this scenario, management practices related to M&A in the banking industry of Pakistan may be more crucial for M&A performance.

Third, different internal and external environmental forces (for instance, state policies, labor unions, and trade unions) may affect the ability of acquirers to implement the management practices at the post-integration stage such as financial and non-financial perks, recruiting, turnover and labor dealings (Weber et al. 2012; Weber and Tarba 2010). Therefore, acquirer’s ethnicity and management practices may shake the capabilities to develop synergy from M&A deal. This study enhances our understanding regarding various management practices use at the integration stage following by M&A. Such management practices may support to cool down the soft issues linked with M&A.

## Proposed management practices

Every M&A transaction is unique in its nature, context, and objectives. To enhance the M&A performance, following management strategies are proposed.

### Coordination approach

As per coordination theory, management of interdependent activities (Jarzabkowski et al. 2012) during M&A is highly significant. An effective coordination among employees and between employer and employees plays a significant role to combine two or more than two organizations into a single entity. Frequent mutual interaction, joint training, and coaching sessions among transition teams are very helpful for the newborn organization (Bohlin et al. 2000) to minimize the soft issues. With reference to synergy theory, through coordination approach employees interact with each other and increase their mutual understanding and synergies (Basile et al. 2012) that not only help to overcome uncertainty, stress, and trauma in their minds but also increase the M&A performance (Ronneberg 2012). Effective coordination approach enhances the coherence among employees and M&A performance (Kale and Singh 2016). Authors suggested that coordination approach as an effective strategy to solve the soft issues and enhance the M&A performance (Gopinath and Becker 2000; Knilans 2009; Zheng et al. 2016). Therefore, it is proposed that;

#### **H1 (a)**

Coordination approach has an effect on M&A FP.

#### **H1 (b)**

Coordination approach has an effect on M&A NFP.

### Coping programs

M&A process creates physical, behavioral and psychological changes and these changes generate stress, trauma, tension, insecurity, and demotivation (Sinkovics et al. 2011) among employees. In such a situation, it is essential for the management and leadership to manage these uncertain situations during M&A process with effective coping programs (Ahammad et al. 2016) which include individual motivation, counseling, training and diagnostic surveys (Musharraf 2003). To design an effective coping program, it is imperative to have a microscopic view of the real situation and access the root causes and intensity of employees’ dysfunctional attitude. Scholars explain different suitable coping programs to enhance the M&A performance (Cording et al. 2014). Employees’ engagement from beginning to the end of M&A process through effective coping program increases the M&A performance (Atkinson and Gary 2016). Several authors conclude that coping programs help to enhance the M&A performance (Amiot 2006; Gunkel et al. 2015). Thus, it is proposed that,

#### **H2 (a)**

Coping programs have an effect on M&A FP.

#### **H2 (b)**

Coping programs have an effect on M&A NFP.

### Communication system

It is difficult for acquirer or merger partners to extract maximum benefits associated with the M&A transaction until the clear and complete M&A plan communicate to the employees (Cording et al. 2014). An effective communication system free from practices like ‘no secret information’, surprise news, propaganda and fake promises may help to make M&A deal more successfully. An active communication system and supported coordination mechanism not only increases the M&A performance but also decreases the stress, tension, and uncertainty among employees (Wan 2015). Open and trustworthy communication system among all stakeholders (e.g. Suppliers, employees, and customers) increases the M&A performance (Osarenkhoe and Hyder 2015). With reference to equity theory, organizational justice theory, and expectancy theory, a clear, timely message propagated with consistency through multiple channels can be helpful to address the employees’ expectation and tensions (Huczynski and Buchanan 2008). Affective, rational and fast communication system inside and outside the organization is recommended by several authors as a strategy to enhance the M&A performance (Appelbaum et al. 2009; Knilans 2009; Zagelmeyer et al. 2016). Thus, it is proposed that;

#### **H3 (a)**

Communication system has an effect on M&A FP.

#### **H3 (b)**

Communication system has an effect on M&A NFP.

### Integration strategy

In spite of having coping programs and better justice perceptions, 80 % organizations are failed at all stages of M&A due to lack of effective integration plan (Atkinson and Gary 2016; Tetenbaum 1999). Several authors explain that effective integration plan has a high impact on M&A performance (Bauer et al. 2016). Successful integration means the emergence of two entities as a one synergized unit. Scholars conclude that integration activities lead to cost saving and a reduction of needed resource which also supports the low transaction cost theory (Bauer et al. 2016; Homburg and Bucerius 2005). There are several aspects of integration, for instance, integration of processes and operations related to HR, culture, finance, marketing, and identity. Authors recommended that an effective integration approach (Schuler and Jackson 2001) is important to increase the M&A performance. Scholars explain that integration strategy has a significant effect on M&A performance (Warter and Warter 2015; Weber et al. 2012). Thus, it is hypothesized that;

#### **H4 (a)**

Integration strategy has an effect on M&A FP.

#### **H4 (b)**

Integration strategy has an effect on M&A NFP.

### Procedural justice

Organizational justice theory originated from equity theory offers a conceptual lens to understand psychological attitudes of employees in response to managerial and strategic decisions like M&A (De Roeck and Swaen 2010). Justice perception, transparency and performance-based decisions supported by accurate data can eliminate dysfunctional reactions and attitudes of employees (Gunkel et al. 2015; Schweiger et al. 1987; Wan et al. 2012). Procedural justice is a significant antecedent of effective merger commitment at pre and integrations stages of M&A (Bebenroth and Thiele 2015). When rational decisions regarding functions, processes, and people are made and affected employees are treated with self-respect, integrity, and care employees’ dysfunctional attitude toward M&A turned into a positive reaction and performance (Ismail and Bebenroth 2016). Procedural justice as a strategy to address the soft issues during M&A has a positive effect on M&A performance (Gunkel et al. 2015; Ismail and Bebenroth 2016; Lipponen et al. 2004). Thus, it is proposed that;

#### **H5 (a)**

Procedural justice has an effect on M&A FP.

#### **H5 (b)**

Procedural justice has an effect on M&A NFP.

### Mediating role of psychological contract

**Fig. 1:**
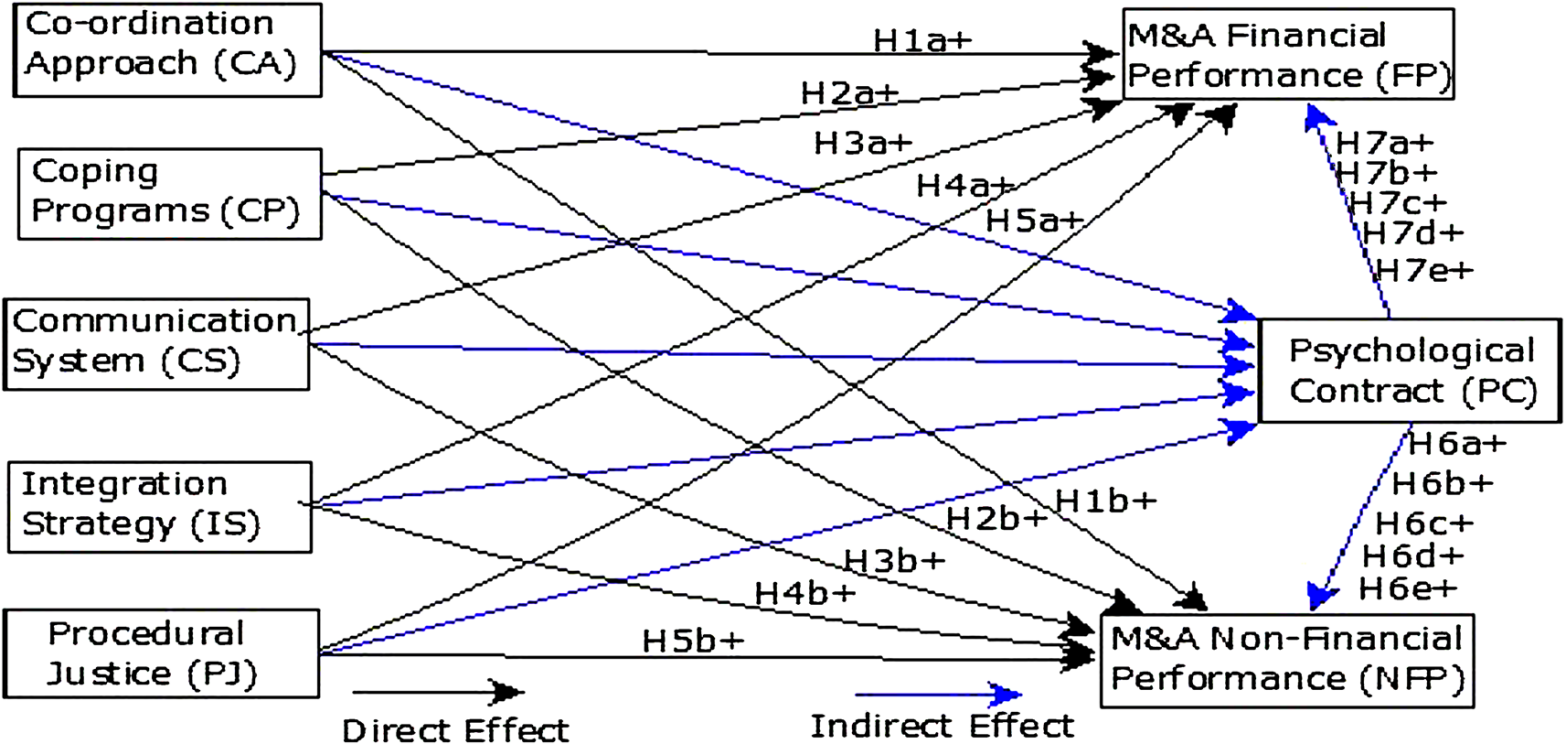
Theoretical framework

According to social exchange theory, by considering the perceived benefits for all parties (acquirer, merger partners or employees) of two business entities which enter into a relationship, it is essential to understand the nature of PC that shapes the attitudes and behaviors at work (Cording et al. 2014). Rousseau categorizes PC into two dimensions, transactional PC, and relational PC (Rousseau 1998). In transactional PC, the relationships are clearly defined and stated without any implied expectations as contract is based on explicit performance outcomes, constrained diversity, and time bound (Yan and Zhu 2013), whereas relational PC is encompassed on trust, care, respect, and justice which is not limited to exchange of economic resources rather it is also pertinent for the exchange of personal and socio-emotional resource (Callea et al. 2016; Lee and Liu 2009). When implied obligations by both parties (employer and employee) remained unfulfilled due to lack of trust and justice, the emergence of HR and culture related issues are certain (Bellou 2007). Fulfillment of PC by employer plays a significant role in overcoming the effects on employees and M&A performance positively (Van den Heuvel et al. 2016). Therefore, HR and management practices proposed to increase M&A performance can be more effective in the presence of fit-PC and high trust level (Erickson 2016; Musharraf 2003). A changing and dynamic environment in organizations during the process of M&A, the role of PC become more significant (Akhtar et al. 2015; Bellou 2007) and may increase the M&A performance (Fig. 1). Thus, it is proposed that;

#### **H6**

PC mediates the effect of each proposed strategy on M&A FP (H6-a, H6-b, H6-c, H6-d, and H6-e).

#### **H7**

PC mediates the effect of each proposed strategy on M&A NFP (H7-a, H7-b, H7-c, H7-d, and H7-e).

### Research model

Following is the research model of the analyzed problem.

- Financial performance (direct effect)
  FP: β_0_ + β_1_ CA + β_2_ CP + β_3_ CS + β_4_ IS + β_5_ PJ +µ
- Financial performance (mediating effect of PC)
  FP: β_0_ + β_1_ CA*PC + β_2_ CP*PC + β_3_ CS*PC + β_4_ IS*PC + β_5_ PJ*PC +µ
- Non-financial performance (direct effect)
  NFP: β_0_ + β_1_ CA + β_2_ CP + β_3_ CS + β_4_ IS + β_5_ PJ +µ
- Non-financial performance (mediating effect of PC)
  NFP: β_0_ + β_1_ CA*PC + β_2_ CP*PC + β_3_ CS*PC + β_4_ IS*PC + β_5_ PJ*PC +µ

FP = Financial Performance, NFP = Non-Financial Performance, CA = Coordination Approach, CP = Coping Program, CS = Communication System, IS = Integration Strategy, PJ = Procedural Justice, PC = Psychological Contract

### Research process

**Fig. 2:**
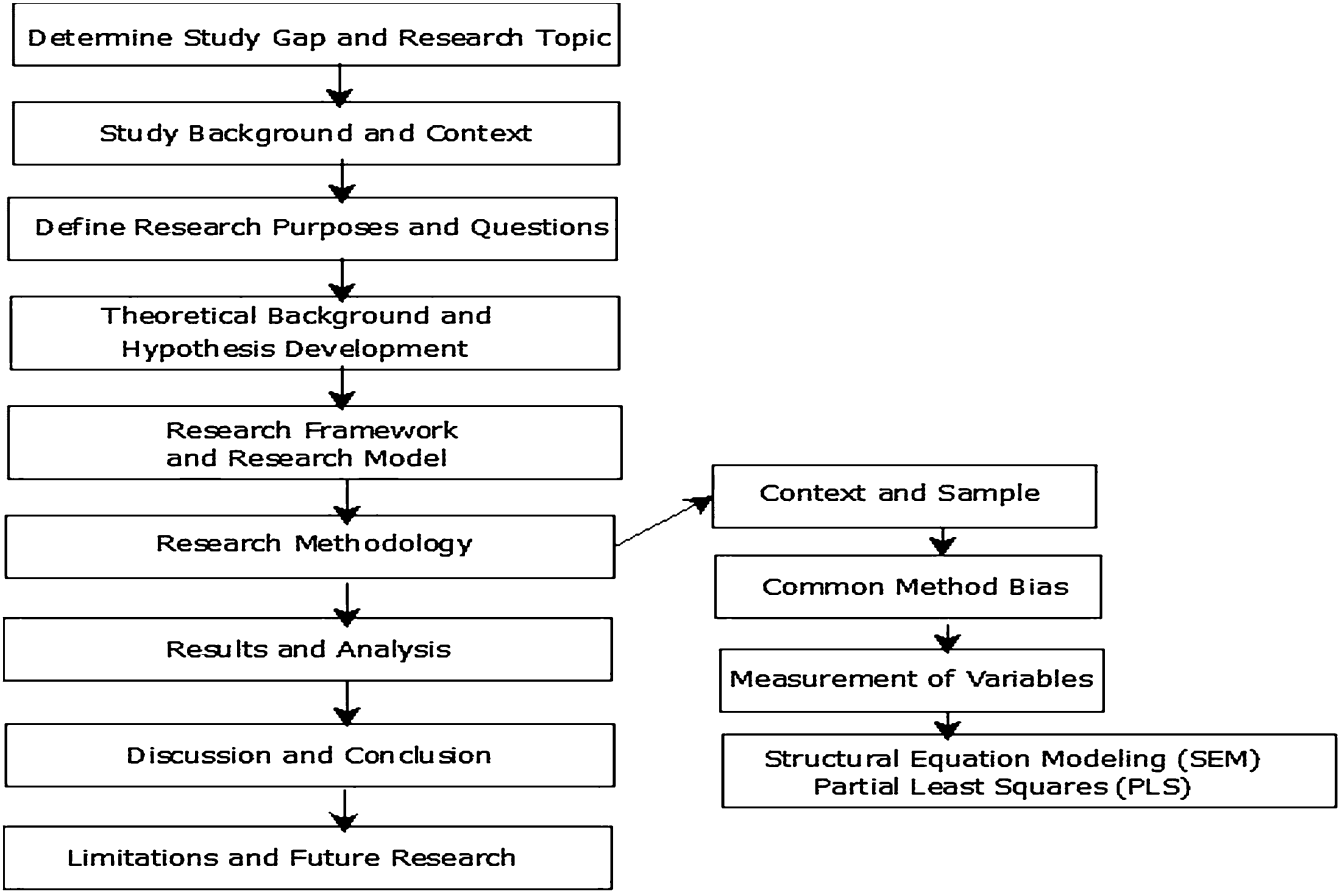
Research flowchart

The present study follows scientific methods during this research process. Research flowchart (Fig. 2) explains all important steps and their sequence which we follow during this study work.

## Research methodology

### Context and sample

In Pakistan, the banking sector has observed more M&A deals as compared to other service sectors; therefore, this sector is selected for the current study. The other rationale for the selection banking sector is to unveil breach of PC in a highly formalized sector of a country. The main reasons behind the PC breach are feeble and expensive judicial system, lack of employment and labor protection laws, unprincipled HR practices and weak bargaining position of employees due to highly imbalance job market (Abbas et al. 2014).

To seek the goals of the present study, we selected 15 commercial banks that observed and experienced the whole process of M&A transactions from 2002 to 2011. The population of this study consists of about 69,231(Banking survey 2013 by KPMG, Karachi, Pakistan) employees in 15 banks, which is located in different area of Pakistan. We selected three big cities (Karachi, Islamabad, and Lahore) to conduct this study survey because some small banks do not have a branch network in local cities and secondly, these three cities are considered main economic, business and social zones and high populated cities of Pakistan. We collected the data in the field. By considering that English is a secondary language of bank employees, we prepared a bilingual questionnaire (English and Urdu) to make understandable for the respondents.

Through “purposive sampling” technique, we selected 700 (“Appendix 1”) bank employees from different management cadre as a sampling unit who observed and experienced the whole process of M&A transactions regardless of their gender, education, designation, and experience. Since a researcher of this study has 7 years’ experience as a bank employee along with one merger and one acquisition transaction experience, therefore, access to sample unit (employees) has not been an issue. Through administrative survey technique, we briefed to each individual regarding the study’ objectives and after approval and consent of that individual, a questionnaire was given to him/her. From 700 respondents, 620 sent back/handover filled questionnaires followed by one soft reminder. After initial screening, from 620 questionnaires 536 were completed and properly filled. Thus, the useable response for the study was 76 %.

### Common method bias

We adopted classical survey procedure and statistical control technique to minimize the common method bias issues (Chung et al. 2015). After statistical analysis (factor analysis), we found that there are no common method bias issues in the data set.

### Measurement of variables

All constructs are measured through structured adapted questionnaire derived from different prior studies with some modification by considering the study objectives and context. The following items are designed to measure employees’ perceptions during M&A process.

#### Coordination approach (CA)

To what extent do you agree or disagree with the following statements? The items under this construct are adopted from the scales developed by (Musharraf 2003). The scale consisted of 03 items in a five-point Likert scale where “1” linked to “strongly disagree” and “5” linked to “strongly agree”. High average scores correspond to high levels of CA. Cronbach’s alpha value is 0.806.

#### Coping program (CP)

To what extent, your company used the following programs during the merger process? The items under this construct are adopted from the scales developed by (Musharraf 2003). The scale consisted of 05 items in a five-point Likert scale where “1” corresponded to “Not use at all” and “5” corresponded to “Used very often”. High average scores correspond to high levels of CP. Cronbach’s alpha value is 0.859.

#### Communication system (CS)

To what extent, your company used the following communication media and plans? The items under this construct are adopted from the scales developed by (Gopinath and Becker 2000; Musharraf 2003). The scale consisted of 04 items in a five-point Likert scale where “1” linked to “Not use at all” and “5” linked to “Used very often”. High average scores correspond to high levels of CS. Cronbach’s alpha value is 0.789.

#### Integration strategy (IS)

To what extent you support to the following items? The items under this construct are adopted from the scales developed by (Musharraf 2003). The scale consisted of 05 items in a five-point Likert scale where “1” linked to “Not use at all” and “5” linked to “Used very often”. High average scores correspond to high levels of IS. Cronbach’s alpha value is 0.864.

#### Procedural justice (PJ)

To what extent do you agree or disagree with the following statements? The items under this construct are adopted from the scales developed by (Gopinath and Becker 2000; Musharraf 2003). The scale consisted of 03 items in a five-point Likert scale where “1” linked to “strongly disagree” and “5” linked to “strongly agree”. High average scores link to high levels of PJ. Cronbach’s alpha value is 0.918.

#### Psychological contract (PC)

The items under this construct are adopted from the scales developed by Wu and Chen (2015). The scale consisted of 05 items scale (two items relate to transactional dimension and three belong to relational dimension) in a five-point Likert scale where “1” corresponded to “strongly disagree” and “5” corresponded to “strongly agree”. High average scores correspond to high levels of PC. Cronbach’s alpha value is 0.806.

#### M&A financial performance (FP)

The items under this construct are adopted from the scales developed by (Musharraf 2003). The scale consisted of 05 items in a five-point Likert scale where “1” corresponded to “strongly disagree” and “5” corresponded to “strongly agree”. This construct has three dimensions (growth in sales, stock price, and market share). High average scores correspond to high levels of FP. Cronbach’s alpha value is 0.908.

#### M&A non-financial performance (NFP)

The items under this construct are adopted from the scales developed by (Musharraf 2003). The scale consisted of 05 items in a five-point Likert scale where “1” corresponded to “strongly disagree” and “5” corresponded to “strongly agree”. This construct has five dimensions (Employees’ satisfaction, customers’ satisfaction, company image in public, operational efficiency and quality of service). High average scores correspond to high levels of NFP. Cronbach’s alpha value is 0.908.

### Partial least squares (regression)

Partial least squares (PLS) is a popular multivariate technique that is used to study complex research models (Hartmann et al. 2010). The PLS based structural equation modeling (SEM) technique is the most appropriate approach for a complex research model as used in this study (Claes Fornell and Larcker 1981; Hartmann et al. 2010; Ringle et al. 2015). Like in this study, in the first phase, a direct relationship between five exogenous variables and two endogenous variables is investigated through 30 constructs and in the second phase, mediating effect (PC) is also observed between exogenous variables and endogenous variable with total 35 constructs. PLS is more appropriate for relatively small sample size (Ali and Park 2016; Rahman et al. 2015). Hypotheses of this study are measured at perceptual response which also recommends PLS-SEM (Gefen et al. 2000). PLS is helpful not only to predict but also explain the variance between key constructs of the study. Another strong benefit of PLS-SEM model is to deal with circumstances about the rustication of knowledge in the case of the distribution of latent variables (Fornell and Cha 1994).

## Results and analysis

This study based on two steps for analysis and interpretation of PLS-SEM based statistical results. At first step reliability and validity of the model is measured and at second phase verification of the structural model and hypotheses is performed.

### Reliability and convergent validity

Reliability test is used to measure the factors consistency, whereas convergent validity is known as a degree, in which all included multiple items are measured at the same concept (Surienty et al. 2014). Outer loading values of all constructs are within range and significant at 0.05 % level of significance. Composite reliability (CR) measures the internal consistency reliability of the model. Table 1 depicts that all values of CR are greater than from the threshold level of 0.70 (Mihail and Kloutsiniotis 2015). All constructs are also meeting the minimum threshold level of 0.70 for Cronbach’s Alpha which is another test to measure the internal consistency reliability of the model (Mihail and Kloutsiniotis 2015). The values of average variance extracted (AVE) are within 0.617–0.736 and these are above the threshold of 0.500 (Bagozzi et al. 1991; Yap et al. 2012). All constructs and their measurements are provided in Table 1.

**Table 1:** Reliability and validity measurement (reflective)

Constructs	Items	LV	CR	α	AVE	SQRT (AVE) > Corr^2^
Communication System	CS-1	0.662	0.865	0.789	0.617	0.79 > 0.68
	CS-2	0.850				
	CS-3	0.812				
	CS-4	0.804				
Coping Programs	CP-1	0.775	0.898	0.859	0.639	0.80 > 0.69
	CP-2	0.835				
	CP-3	0.836				
	CP-4	0.834				
	CP-5	0.709				
Coordination Approach	CA-1	0.833	0.886	0.806	0.721	0.85 > 0.83
	CA-2	0.865				
	CA-3	0.849				
Integration Strategy	IS-1	0.833	0.902	0.864	0.650	0.81 > 0.79
	IS-2	0.814				
	IS-3	0.868				
	IS-4	0.700				
	IS-5	0.807				
Procedural Justice	PJ-1	0.897	0.893	0.819	0.736	0.86 > 0.82
	PJ-2	0.795				
	PJ-3	0.878				
Psychological Contract	PC-1	0.834	0.923	0.896	0.706	0.84 > 0.81
	PC-2	0.825				
	PC-3	0.820				
	PC-4	0.872				
	PC-5	0.851				
Financial Performance	FP-1	0.850	0.931	0.908	0.736	0.86 > 0.84
	FP-2	0.859				
	FP-3	0.864				
	FP-4	0.870				
	FP-5	0.831				
Non-Financial Performance	NFP-1	0.728	0.932	0.908	0.734	0.86 > 0.77
	NFP-2	0.892				
	NFP-3	0.891				
	NFP-4	0.894				
	NFP-5	0.865				

All loadings are significant at 0.050 level (2-tailed)

*α* = Cronbach’s alpha

*LV* loading values,*CR*  composite reliability, *AVE* average variance extracted, *Corr2* highest squared correlation between constructs

### Discriminant validity

Discriminant validity of all reflective constructs in the model is measured by two approaches, Fornell–Lacker criterion, and cross-loadings. “Appendix 2” indicates that cross-loadings of item’s outer loading to the linked variables are higher than all of its loading values of other variables (Mihail and Kloutsiniotis 2015). As per Fornell–Lacker criterion, the square root of each AVE is compared to the correlation of all constructs along with their items and confirmed that all AVE square root are higher than the correlation values (Ali and Park 2016; Mihail and Kloutsiniotis 2015). The detail measurements through Fornell–Lacker criterion is given in Table 2.

**Table 2:** Discriminant validity

	CA	CP	CS	FP	IS	NFP	PC	PJ
CA	*0.849*							
CP	0.671	*0.799*						
CS	0.652	0.645	*0.785*					
FP	0.790	0.661	0.671	*0.855*				
IS	0.839	0.695	0.676	0.773	*0.806*			
NFP	0.762	0.655	0.683	0.841	0.750	*0.857*		
PC	0.794	0.671	0.653	0.824	0.797	0.777	*0.840*	
PJ	0.788	0.613	0.613	0.792	0.778	0.759	0.824	*0.858*

Italic values indicate significance level of p value (p < 0.05)

Robustness of the model and Collinearity issues in the data are verified through Variance Inflation Factor (VIF) (“Appendix 3”). The values of VIF ensure the results of the model for policy implication. Collinearity issues are not found in the data as all VIF values are <05 (Hair et al. 2013, 2014b). Further verification of structural model, R^2^, and Q^2^ techniques are used. The R^2^ value of each dependent variable is a degree of the variance explained in each dependent variable and predictive accuracy of the model. The rule of thumb is R^2^ values ≥0.75; ≥0.50 and ≥0.25 are considered substantial, moderate, and weak respectively (Chin 1998; Hair et al. 2014a, b; Mihail and Kloutsiniotis 2015). R^2^ values of endogenous variables (PC, FP, and NFP) are 0.767, 0.760 and 0.708 respectively that indicate a good strength of structural model (Ali and Park 2016). Blindfolding technique (SmartPLS-3) is used for Q^2^ calculation. After two times application of blindfolding technique at omission distance 7 and 25, the results of Q^2^ were stable and considerably above from zero (Henseler and Sarstedt 2013; Mihail and Kloutsiniotis 2015). The positive and significant results of R^2^ and Q^2^ are strong evidence about the quality and strength of structured model (Ali and Park 2016).

### Hypotheses verification (direct relationship)

The results reported in the Table 3 indicate that CA, CS, PJ have a direct significant effect on M&A FP which confirms H1-a, H3-a, and H5-a. However CP, IS has not direct significant effect on M&A FP which does not support our hypothesis H2-a, and H4-a. In contrast, CA, CP, CS, PJ have a direct effect on M&A NFP which supports hypothesis H1-b, H2-b, H3-b, and H5-b. However, IS has not a significant effect on M&A NFP and does not support hypothesis H4-b.

**Table 3:** Hypotheses verification (direct relationship)

Structural path	Path coefficient (t-value)	Effect size (f^2^)	Confidence Interval (95 %)	(*p* value) 0.05 %	Results
CA → FP	0.188 (3.244)	0.034	(0.087–0.278)	0.001	Supported (H1-a)
CP → FP	0.061 (1.676)	0.007	(0.004–0.120)	0.094	Not Supported (H2-a)
CS → FP	0.120 (3.639)	0.028	(0.067–0.173)	0.000	Supported (H3-a)
IS → FP	0.063 (1.133)	0.004	(−0.033–0.153)	0.256	Not Supported (H4-a)
PJ → FP	0.200 (3.669)	0.043	(0.105–0.287)	0.000	Supported (H5-a)
CA → NFP	0.178 (2.865)	0.025	(0.067–0.269)	0.004	Supported (H1-b)
CP → NFP	0.083 (2.003)	0.011	(0.003–0.150)	0.045	Supported (H2-b)
CS → NFP	0.188 (5.015)	0.056	(0.128–0.251)	0.000	Supported (H3-b)
IS → NFP	0.075 (1.265)	0.004	(−0.013–0.177)	0.206	Not Supported (H4-b)
PJ → NFP	0.206 (3.837)	0.038	(0.115–0.290)	0.000	Supported (H5-b)
CA → PC	0.169 (3.181)	0.029	(0.089–0.265)	0.001	
CP → PC	0.109 (3.173)	0.023	(0.054–0.168)	0.002	
CS → PC	0.080 (2.230)	0.013	(0.022–0.139)	0.026	
IS → PC	0.191 (3.826)	0.036	(0.102–0.266)	0.000	
PJ → PC	0.430 (8.939)	0.261	(0.352–0.511)	0.000	
PC → FP	0.344 (5.486)	0.115	(0.249–0.458)	0.000	
PC → NFP	0.227 (4.027)	0.041	(0.142–0.329)	0.000	

### Hypotheses verification (indirect relationship)

For the verification of mediating effect, bootstrapping technique (SmartPLS-3) is used through 3000 randomly drawn samples with replacement at 0.05 % level of significance (Hair et al. 2014b; Ringle et al. 2015). In Table 4, three different levels of mediating effects of five exogenous constructs are verified. (1) CA has partial mediation effect on M&A FP through PC and support H6-a, however, no mediation effect on M&A NFP and does not support H7-a. (2) CP has an indirect effect on M&A FP and NFP through PC and supports H6-b and H7-b. (3) CS does not have an indirect effect on M&A FP and NFP through PC and does not support H6-c and H7-c. (4) IS has an indirect effect on M&A FP and NFP through PC and supports H6-d and H7-d. (5) PJ has partial mediation effect on M&A FP and NFP through PC and supports H6-e and H7-e.

**Table 4:** Hypotheses verification (indirect relationship)

Effect of	Direct effect (t-value)	Indirect effect (t-value)	Total effect	VAF (%)	Interpretation	Results
CA → PC → FP	0.188 (3.244)	0.058 (2.566)	0.246	23.56	Partial mediation	Supported (H6-a)
CP → PC → FP	0.061 (1.676)	0.038 (2.761)	0.098	38.77	Indirect mediation	Supported (H6-b)
CS → PC → FP	0.120 (3.639)	0.027 (1.935)	0.147n.s	18.36	No mediation	Not supported (H6-c)
IS → PC → FP	0.063 (1.133)	0.066 (3.367)	0.129	51.16	Indirect mediation	Supported (H6-d)
PJ → PC → FP	0.200 (3.669)	0.148 (4.620)	0.348	42.52	Partial mediation	Supported (H6-e)
CA → PC → NFP	0.178 (2.865)	0.038 (2.431)	0.216n.s	17.59	No mediation	Not supported (H7-a)
CP → PC → NFP	0.083 (2.003)	0.025 (2.343)	0.108	23.14	Partial mediation	Supported (H7-b)
CS → PC → NFP	0.188 (5.015)	0.018 (1.769)	0.206n.s	08.73	No mediation	Not supported (H7-c)
IS → PC → NFP	0.075 (1.265)	0.043 (2.973)	0.118	36.44	Indirect mediation	Supported (H7-d)
PJ → PC → NFP	0.206 (3.837)	0.098 (3.585)	0.303	32.34	Partial mediation	Supported (H7-e)

*VAF* variance accounted for, *n.s* not significant; |t| ≥ 1.96 at p = 0.05 level; The VAF > 80 % indicates full mediation, 20 % ≤ VAF ≥ 80 % shows partial mediation while VAF < 20 % as no mediation (Ali and Park 2016)

In literature, there is no covenant yet that the relationship between independent and dependent variables can be significant excluding defined mediator (Ali and Park 2016; Zhao et al. 2010). However, it is a condition that indirect effect through mediator has to be significant and mediation is creating a significant effect on endogenous variables by fascinating some indirect effect of exogenous variables (Ali and Park 2016; Hair et al. 2014b). The present paper used non-parametric bootstrapping approach to measuring the significant level of mediating effect (Ali and Park 2016). In Table 4, “variance accounted for” is used to recognize the indirect effect size with reference to the total effect (Hair Jr et al. 2014a).

## Discussion and conclusion

**Fig. 3:**
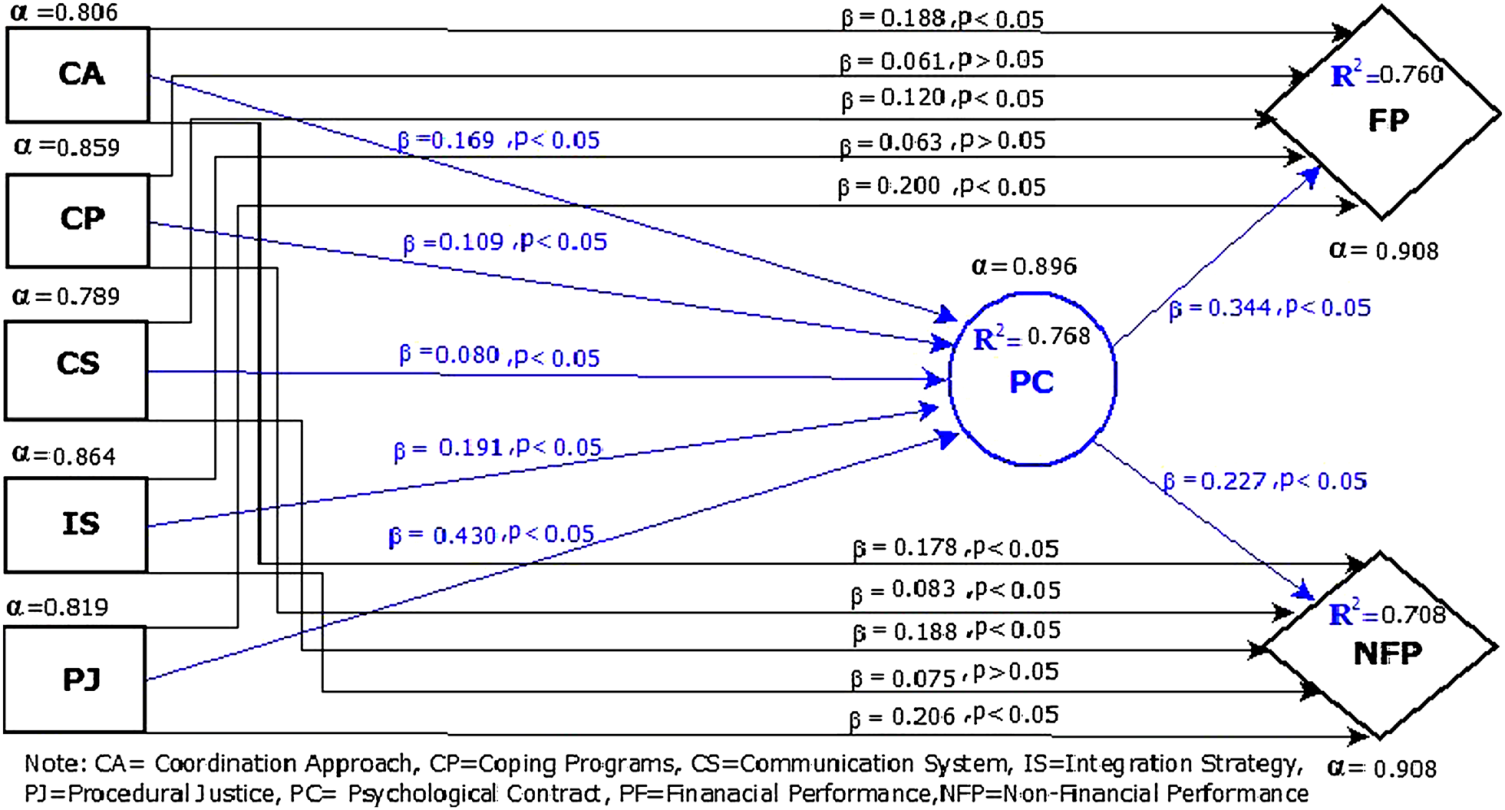
Structural model

Recently, different scholars and M&A consultants through their studies and reports confirm that organizations are trying hard to improve management practices to solve the soft issues, leading to increasing in M&A performance (Brueller et al. 2016; Dixon 2005; Jayadev and Sensarma 2007; Kale and Singh 2016). Management strategies (CA, CP, CS, IS, and PJ) can be a good tool to enhance the M&A performance as they are considered accommodating to solve the soft issues during integration stage following by M&A. This study enhances the understanding of the soft issues related to M&A phenomena by investigating the relationship between management practices (CA, CP, CS, IS, and PJ) and M&A FP and NFP. This relationship contributes to the social exchange theory, social interaction theory (Yang and Wang 2011), organizational justice theory and theory of absorptive capacity by investigating the mediating role of PC theory (Rousseau 1998) between said management practices and M&A performance. The complete nomological network is placed on the study framework along with statistical results (Fig. 3). The PC as a mediator partially mediates the association among CA to M&A FP, CP to M&A NFP, and PJ to M&A FP and NFP. However, CP to M&A FP, IS to M&A FP and NFP have an indirect effect which highlights the effective role of PC theory in M&A performance at post integration stage. But PC does not mediate the effect of CS on M&A FP and NFP. According to the “problematic integration theory”, people (employees) perceive, consider, evaluate and counter to the information according to their social and cultural structure and environment (Babrow 2001). As discussed before that Pakistan has weak corporate structure, feeble judicial system and unstable economic and political environment which may be a reason behind this distrust of employees on management and its communication system.

M&A is a process of changes which creates uncertainty, stress and ambiguous situation among employees. A context (Pakistan) where the feeble judicial system, lack of professionalism and imbalance job market prevails, it is not tranquil to believe on the communication system and easy to perceive PC breach (Syed 2010). Considering the recommended study by Musharraf (Musharraf 2003), this study developed a research framework that interlinks management practices (CA, CP, CS, IS, PJ), and M&A FP and NFP. Previous studies (Garrow 2005; Musharraf 2003) classify that said strategic mechanism increase M&A performance, provided that, a positive trusted culture should be maintained between employer and employees during M&A. Findings of this study signify that a positive PC can enhance the effectiveness of said management practices to increase the M&A performance (Mao and Liu 2009; Rousseau 1998), subject to the context of the organizations that involved in M&A which is also confirmed by previous studies (Edwards and Edwards 2012; Papadakis 2005).

M&A strategies are adopted to enhance the volume and financial strength of businesses (Buckley and Ghauri 2002). Therefore, Organizations are more focused on apparently direct finance related issues (hard issues) to achieve the financial objective, however; success rate is 30–40 % (Schneider 2008). Recently, different studies pointed out those soft issues are the significant cause behind this low achievement rate (Bauer et al. 2016; Meyer 2001; Weber and Tarba 2010). The said management practices are not only helpful to solve these soft issues, but also increase the M&A performance. Since the empirical investigation of this study ascertains that PC accelerates the effectiveness of these strategies to solve the soft issues and enhances the M&AFP and NFP. The significant point in this study increases in FP higher than NFP. It is also in line with the previous studies (Musharraf 2003; Schuler and Jackson 2001). Increase in NFP is also indirectly support to the M&A FP (Spencer et al. 2009). From five exogenous variables, PJ is a most effective strategy which directly and through PC positively enhances the M&A performance. With reference to the organizational justice theory, when employees feel a sense of injustice by the organization side they negatively change their attitude and productivity level (Baldwin 2006). In reality, soft issues are real hard ones (Pikula 1999), which really need to solve as an indirect effective solution of hard issues and increase in M&A performance. The role of PC is significant to enhance the M&A performance through management practices in the banking industry of Pakistan.

This is one of the PLS-SEM based empirical studies which assess the mediating role of PC in M&A context of Pakistan banking industry. The results of this study do not only support the previous research findings but also agree with the latest literature on management practices, psychological contract, and M&A performance. This study indicates some significant understandings and implications for professional bankers, M&A consultants and leadership of banking organizations not only for Pakistan but also other developing countries especially south Asian countries.

This study recommends that enquirers’ management and consultants may solve soft issues through management practices before and after entering into the M&A process. Once these soft issues are solved, employees will be motivated positively for M&A process and hard issues (financial, legal and marketing issues) will be solved easily. As in other developing countries, Pakistan has the weak judicial system not only at the state level but also at a corporate level which develops a sense of uncertainty and insecurity among employees regarding their rights and future career. PJ is highly impactful strategy and management practice that proved in this study. Therefore, it is suggested that management should realize to the employees about strong and fair justice system within an organization. Once the sense of justice will prevail within the organization, the trust level at organization’s communications system will automatically develop.

## Limitations and future research

Like other studies, this study also has some limitations which offer further research roadmaps. First, the sample size of the present study is comparatively small (total population) which may bound the validity of the present paper. Second, the present study design is based on a cross-sectional assessment which collects data/employees feedback from a particular sample to present the larger population at one point in time (Musharraf 2003). Third, the present paper examines the overall impact of culture, and HR related forces in the performance of M&A. However, the present study does not focus the exact impacts of specific elements such as redundancies, favoritism, training programs compensation, and career growth etc., on the performance of M&A. Fourth, the present study investigates the overall mediating role of PC among management practices and M&A performance, however, literature depicts the different impact of relational PC and transactional PC on M&A performance (Callea et al. 2016; Jensen et al. 2009). Fifth, although, we used varied techniques to increase the survey returns of this study, however, this study still undergoes from the problem of a comparatively modest sample size. This issue can be solved by using other data collection techniques such as personal interviews, open-ended questionnaires etc.

In future research, the role of relational PC and transactional PC in the performance of M&A should be investigated separately. This separation will be helpful to determine that which dimension of PC is more effective as a mediator. Secondly, the impact of three M&A stages (Pre, Integration, and Post) separately as a moderator on PC may be investigated. Thirdly, comparative studies between countries and industries are recommended to evaluate the role of management practices in the performance of M&A.

## Appendix 1

See Table 5.

**Table 5:** Demographic profile, N = 536

Variables	Category	Frequency	Proportions (%)
Gender	Male	345	64
	Female	191	36
Age	20–35 years	177	33
	36–50 years	225	42
	51 and above	134	25
Experience	Less than 10 years	191	36
	10–20 years	237	44
	More than 20 years	108	20
Job position	Lower	215	40
	Middle	247	46
	Upper	074	14
Education	Inter college (12 years education)	079	15
	Graduation (14 years education)	228	42
	Masters (16 years education)	161	30
	M Phil (18 years education)	068	13

## Appendix 2

See Table 6.

**Table 6:** Factor loadings (cross loadings) of measurement model

	CA	CP	CS	FP	IS	NFP	PC	PJ
CA-1	*0.833*	0.598	0.578	0.655	0.705	0.657	0.640	0.654
CA-2	*0.865*	0.593	0.563	0.662	0.721	0.634	0.714	0.845
CA-3	*0.849*	0.515	0.516	0.695	0.714	0.651	0.667	0.723
CP-1	0.423	*0.775*	0.468	0.447	0.434	0.445	0.449	0.396
CP-2	0.465	*0.835*	0.486	0.500	0.503	0.500	0.507	0.462
CP-3	0.463	*0.835*	0.478	0.493	0.473	0.473	0.501	0.489
CP-5	0.461	*0.835*	0.526	0.498	0.515	0.472	0.495	0.494
CP-5	0.759	*0.709*	0.575	0.638	0.752	0.654	0.661	0.667
CS-1	0.489	0.499	*0.662*	0.535	0.564	0.554	0.532	0.498
CS-2	0.533	0.506	*0.850*	0.538	0.512	0.529	0.533	0.521
CS-3	0.511	0.542	*0.812*	0.523	0.538	0.554	0.512	0.509
CS-4	0.499	0.467	*0.804*	0.496	0.493	0.495	0.459	0.465
FP-1	0.682	0.601	0.568	*0.850*	0.677	0.715	0.728	0.679
FP-2	0.695	0.604	0.600	*0.859*	0.669	0.735	0.720	0.679
FP-3	0.671	0.552	0.580	*0.864*	0.667	0.706	0.705	0.694
FP-4	0.683	0.539	0.554	*0.870*	0.645	0.721	0.701	0.685
FP-5	0.643	0.528	0.560	*0.831*	0.648	0.721	0.682	0.666
IS-1	0.605	0.629	0.581	0.590	*0.833*	0.577	0.592	0.591
IS-2	0.708	0.569	0.551	0.638	*0.814*	0.649	0.713	0.689
IS-3	0.666	0.577	0.541	0.616	*0.868*	0.613	0.628	0.619
IS-4	0.661	0.479	0.461	0.605	*0.700*	0.533	0.539	0.639
IS-5	0.737	0.543	0.579	0.664	*0.807*	0.641	0.724	0.738
NFP-1	0.507	0.458	0.511	0.658	0.565	*0.728*	0.537	0.560
NFP-2	0.703	0.605	0.620	0.748	0.675	*0.892*	0.738	0.694
NFP-3	0.719	0.589	0.605	0.747	0.688	*0.891*	0.705	0.699
NFP-4	0.665	0.586	0.594	0.721	0.635	*0.894*	0.680	0.677
NFP-5	0.648	0.550	0.589	0.728	0.643	*0.865*	0.650	0.655
PC-1	0.663	0.561	0.563	0.705	0.668	0.687	*0.824*	0.769
PC-2	0.634	0.503	0.494	0.629	0.633	0.586	*0.833*	0.655
PC-3	0.587	0.570	0.551	0.636	0.637	0.611	*0.821*	0.603
PC-4	0.684	0.574	0.538	0.725	0.687	0.679	*0.872*	0.697
PC-5	0.752	0.606	0.590	0.767	0.722	0.692	*0.851*	0.728
PJ-1	0.865	0.593	0.563	0.662	0.721	0.634	0.714	*0.897*
PJ-2	0.642	0.542	0.500	0.664	0.685	0.631	0.636	*0.795*
PJ-3	0.674	0.485	0.532	0.665	0.636	0.660	0.712	*0.878*

Italic values indicate significance level of p value (p < 0.05)

## Appendix 3

See Table 7.

**Table 7:** VIF

	CA	CP	CS	IS	PC	PJ
FP	4.310	2.293	2.162	4.487	4.310	3.849
NFP	4.310	2.293	2.162	4.487	4.310	3.849
PC	4.187	2.241	2.135	4.329	–	3.051

## References

[CR1] Abbas Q, Hunjra AI, Azam RI, Shahzad Ijaz M, Zahid M (2014). Financial performance of banks in Pakistan after Merger and Acquisition. J Glob Entrep Res.

[CR2] Ahammad MF, Tarba SY, Liu Y, Glaister KW (2016). Knowledge transfer and cross-border acquisition performance: the impact of cultural distance and employee retention. Int Bus Rev.

[CR3] Ahmad R, Rashid M, Zia-ur-rehman M (2011). Managing post-acquisition cultural change: a case study of union bank limited ( Now Standard Chartered Bank Pakistan Limited ). Int J Trade Econ Finance.

[CR4] Akhtar MN, Bal M, Long L (2016). Exit, voice, loyalty, and neglect reactions to frequency of change, and impact of change: a sensemaking perspective through the lens of psychological contract. Empl Relat.

[CR5] Al Jerjawi K (2011). HR managers’ roles & contributions in merger processes. International Journal of Human Resource Studies.

[CR6] Ali M, Park K (2016). The mediating role of an innovative culture in the relationship between absorptive capacity and technical and non-technical innovation. J Bus Res.

[CR7] Amiot CE (2006). A longitudinal investigation of coping processes during a merger: implications for job satisfaction and organizational identification. J Manag.

[CR8] Appelbaum SH, Roberts J, Shapiro BT (2009). Cultural strategies in M & As: investigating ten case studies. J Exec Educ.

[CR9] Arshad A (2012). Post merger performance analysis of standard chartered bank Pakistan. Interdiscip J Contemp Res Bus.

[CR10] Atkinson S, Gary MS (2016). Mergers and acquisitions: modeling decision making in integration projects. Behav Oper Res.

[CR11] Babrow AS (2001). Uncertainty, value, communication, and problematic integration. J Commun.

[CR12] Bagozzi RP, Yi Y, Phillips LW (1991). Assessing construct validity in organizational research. Adm Sci Q.

[CR13] Baldwin S (2006) Organisational justice. Institute for Employment Studies. Retrieved from http://www.employment-studies.co.uk/system/files/resources/files/mp73.pdf

[CR14] Basile R, Capello R, Caragliu A (2012). Technological interdependence and regional growth in Europe: proximity and synergy in knowledge spillovers. Pap Reg Sci.

[CR15] Bauer F, Matzler K, Wolf S (2016). M&A and innovation: The role of integration and cultural differences—a central European targets perspective. Int Bus Rev.

[CR16] Bebenroth R, Thiele KO (2015) When organizational justice matters for affective merger commitment. Thunderbird International Business Review. Retrieved from http://onlinelibrary.wiley.com/doi/10.1002/tie.21820/full

[CR17] Bellou V (2007). Psychological contract assessment after a major organizational change: the case of mergers and acquisitions. Empl Relat.

[CR18] Bohlin N, Daley E, Thomson S (2000). Successful post-merger integration: realizing the synergies. Handbook of Business Strategy.

[CR19] Bower JL (2001) Not all M & As are alike—and that matters. Harvard Bus Rev 79(3), 93–101. Retrieved from https://books.google.com.pk/books 11246927

[CR20] Brueller NN, Carmeli A, Markman GD (2016). Linking merger and acquisition strategies to postmerger integration: a configurational perspective of human resource management. J Manag.

[CR21] Brush TH (1996). Predicted change in operational synergy and post-acquisition performance of acquired businesses. Strateg Manag J.

[CR22] Buckley PJ, Ghauri PN (2002). International mergers and acquisitions: a reader.

[CR23] Buiter JE, Harris CM (2013). Post-merger influences of human resource practices and organizational leadership on employee perceptions and extra-role behaviors. SAM Adv Manag J.

[CR24] Callea A, Urbini F, Ingusci E, Chirumbolo A (2016). The relationship between contract type and job satisfaction in a mediated moderation model: the role of job insecurity and psychological contract violation. Econ Ind Democr.

[CR25] Campa JM, Hernando I (2005) M&A performance in the European financial industry. Working Paper, vol 3, no 588, pp 1–29

[CR26] Cartwright S, Cooper CL (1993). The role of culture compatibility in successful organizational marriage. Acad Manag Perspect.

[CR27] Cartwright S, Cooper CL (2014). Mergers and acquisitions: the human factor.

[CR28] Cartwright S, Schoenberg R (2006). Thirty years of mergers and acquisitions research: Recent advances and future opportunities. Br J Manag.

[CR29] Chin WW (1998). The partial least squares approach to structural equation modeling. Modern Methods Bus Res.

[CR30] Chung HFL, Yang Z, Huang PH (2015). How does organizational learning matter in strategic business performance? The contingency role of guanxi networking. J Bus Res.

[CR31] Cording M, Harrison JS, Hoskisson RE, Jonsen K (2014). Walking the talk: a multistakeholder exploration of organizational authenticity, employee productivity, and post-merger performance. Acad Manag Perspect.

[CR32] Creasy T, Stull M, Peck S (2009). Understanding employee- level dynamics within the merger and acquisition process. J Gen Manag.

[CR33] De Roeck K, Swaen V (2010) The role of CSR on employees’ post-merger organizational identification. Louvain School of Management Working Paper, pp 1–23

[CR34] De Silva V, Opatha HHDNP (2015) Role of ethical orientation of HRM in establishing an ethical organizational culture: a literature review and implications, pp 1–24

[CR35] Di Guardo C, Harrigan KR, Marku E (2015). Quantity at expense of quality? Measuring the effects of technological M&A on innovation and firm performance. SSRN Electron J.

[CR36] Dixon I (2005) Cultural issues in mergers and acquisitions. Human Resour Manag. Retrieved from http://www2.deloitte.com/content/dam/Deloitte/us/Documents/mergers-acqisitions/us-ma-consulting-cultural-issues-in-ma-010710.pdf

[CR37] Edwards MR, Edwards T (2012). Company and country effects in international mergers and acquisitions: employee perceptions of a merger in three European countries. Econ Ind Democr.

[CR38] Empson L (2001). Fear of exploitation and fear of contamination: impediments to knowledge transfer in mergers between professional service firms. Hum Relat.

[CR39] Erickson RA (2016). Communication and Employee Retention.

[CR40] Fornell C, Cha J (1994). Partial least squares. Adv Methods Market Res.

[CR41] Fornell C, Larcker DF (1981). Structural equation models with unobservable variables and measurement error: algebra and statistics. J Mark Res.

[CR42] Garrow VJ (2005) The psychological contract in the context of mergers and acquisitions. University of London. Retrieved from http://ethos.bl.uk/OrderDetails.do?uin=uk.bl.ethos.421766

[CR43] Gefen D, Straub D, Boudreau MC (2000). Structural equation modeling and regression: guidelines for research practice. Commun Assoc Inf Syst.

[CR44] Giessner SR, Horton KE, Humborstad SIW (2016). Identity management during organizational mergers: empirical insights and practical advice. Soc Issues Policy Rev.

[CR45] Gomes E, Angwin D, Peter E, Mellahi K (2012). HRM issues and outcomes in African mergers and acquisitions: a study of the Nigerian banking sector. Int J Hum Resour Manag.

[CR46] Gopinath C, Becker TE (2000). Communication, procedural justice, and employee attitudes: relationships under conditions of divestiture. J Manag.

[CR47] Gunkel M, Schlaegel C, Rossteutscher T, Wolff B (2015). The human aspect of cross-border acquisition outcomes: the role of management practices, employee emotions, and national culture. Int Bus Rev.

[CR48] Hagedoorn J, Duysters G (2002). External sources of innovative capabilities: the preferences for strategic alliances or mergers and acquisitions. J Manag Stud.

[CR49] Haider A, Shoaib M, Kanwal S (2015). 2015. Impact of mergers on performance of banking sector of Pakistan.

[CR50] Hair JF, Ringle CM, Sarstedt M (2013). Partial least squares structural equation modeling: rigorous applications, better results and higher acceptance. Long Range Plan.

[CR51] Hair F, Sarstedt M, Hopkins L, Kuppelwieser GV (2014). Partial least squares structural equation modeling (PLS-SEM): an emerging tool in business research. Eur Bus Rev.

[CR52] Hair JFJ, Hult GTM, Ringle C, Sarstedt M (2014). A primer on partial least squares structural equation modeling (PLS-SEM). Long Range Plan.

[CR53] Hartmann F, Naranjo-Gil D, Perego P (2010). The effects of leadership styles and use of performance measures on managerial work-related attitudes. Eur Account Rev.

[CR54] Henseler J, Sarstedt M (2013). Goodness-of-fit indices for partial least squares path modeling. Comput Stat.

[CR55] Homburg C, Bucerius M (2005). A marketing perspective on mergers and acquisitions: how marketing integration affects postmerger performance. J Mark.

[CR56] Huczynski AA, Buchanan DA (2008). Organizational behaviour. Annu Rev Psychol.

[CR57] Ismail M, Bebenroth R (2016). Organizational justice and organizational identification of millennials in mergers and acquisitions: a conceptual framework. Eur J Soc Sci.

[CR58] Jarzabkowski PA, Le JK, Feldman MS (2012). Toward a theory of coordinating: creating coordinating mechanisms in practice. Organ Sci.

[CR59] Jayadev M, Sensarma R (2007) Mergers in Indian banking: an analysis. South Asian J Manag 14(4): 20–49. Retrieved from http://hdl.handle.net/2299/3465

[CR60] Jensen JM, Opland RA, Ryan AM (2009). Psychological contracts and counterproductive work behaviors: employee responses to transactional and relational breach. J Bus Psychol.

[CR61] Joash GO, Njangiru MJ (2015) The effect of mergers and acquisitions on financial performance of banks (a survey of commercial banks in Kenya). Int J Innov Res Dev 16, 48–57. Retrieved from www.ijird.com

[CR62] Kale P, Singh H (2016) Management of overseas acquisitions by developing country multinationals and its performance implications: the Indian example. Thunderbird Int Bus Rev. Retrieved from http://onlinelibrary.wiley.com/doi/10.1002/tie.21818/full

[CR63] Kapoor R, Lim K (2007). The impact of acquisitions on the productivity of inventors at semiconductor firms: a synthesis of knowledge-based and incentive-based perspectives. Acad Manag J.

[CR64] Kazík R (2012) The impact of the corporate culture on the success or the failure of mergers and acquisitions, pp 02–20. Retrieved from http://www.opf.slu.cz/aak/2012/04/Kazik.pdf

[CR65] Kemal MU (2011). Post-merger profitability: a case of Royal Bank of Scotland (RBS). Int J Bus Soc Sci.

[CR66] King DR, Dalton DR, Daily CM, Covin JG (2004). Meta-analyses of post-acquisition performance: Indications of unidentified moderators. Strateg Manag J.

[CR67] Knilans G (2009). Mergers and acquisitions: best practices for successful integration. Employ Relat Today.

[CR68] Kouser R, Saba I (2011). Effects of business combination on financial performance: evidence from Pakistan’s banking sector. Aust J Bus Manag Res.

[CR69] Lee H-W, Liu C-H (2009). The relationship among achievement motivation, psychological contract and work attitudes. Soc Behav Personal.

[CR70] Lipponen J, Olkkonen M-E, Moilanen M (2004). Perceived procedural justice and employee responses to an organizational merger. Eur J Work Organ Psychol.

[CR71] Mao H, Liu X (2009) Psychological contract in the process of enterprises’ merger, acquisition and integration. Can Soc Sci 4(1): 22–26. Retrieved from http://50.22.92.12/index.php/css/article/view/339

[CR72] Meglio O, Risberg A (2011). The (mis)measurement of M&A performance—a systematic narrative literature review. Scand J Manag.

[CR73] Meyer CB (2001). Allocation processes in mergers and acquisitions: an organizational justice perspective. Br J Manag.

[CR74] Mihail DM, Kloutsiniotis PV (2015). The effects of high-performance work systems on hospital employees’ work-related well-being: evidence from Greece. Eur Manag J.

[CR75] Musharraf MA (2003) The role of human resource and cultural factors in the success or failure of merger & acquisition strategies: the case of Saudi Arabia, Saudi Arabia. Retrieved from http://etheses.whiterose.ac.uk/4207/

[CR76] Osarenkhoe A, Hyder A (2015). Marriage for better or for worse? Towards an analytical framework to manage post-merger integration process. Bus Process Manag J.

[CR77] Papadakis VM (2005). The role of broader context and the communication program in merger and acquisition implementation success. Manag Decis.

[CR78] Pikula DA (1999) Mergers & acquisitions: organizational culture & HR issues. IRC Press. Retrieved from http://irc.queensu.ca/sites/default/files/articles/mergers-and-acquisitions-organizational-culture-and-hr-issues.pdf

[CR79] Rahman SA, Taghizadeh SK, Ramayah T, Ahmad NH (2015). Service innovation management practices in the telecommunications industry: What does cross country analysis reveal?. SpringerPlus.

[CR80] Ringle C, Wende S, Becker J (2015) SmartPLS 3. Bönningstedt: SmartPLS. Retrieved from http://www.smartpls.com

[CR81] Ronneberg L (2012) Synergy created by coordinating sourcing in related diversified firms, a study of the Norwegian utility industry. Linnaeus. Retrieved from http://www.diva-portal.org/smash/record.jsf?pid=diva2:548184&dswid=-8584

[CR82] Rousseau DM (1998). The “problem” of the psychological contract considered. J Organ Behav.

[CR83] Santander B (2007) Ultimate law guide case study: Royal Bank of Scotland consortium takeover of ABN Amro. Retrieved August 5, 2016, from http://www.ultimatelawguide.com/tl_files/ulg/downloads/commercialawareness/RBStakeoverofABNAmro.pdf

[CR84] SBP (2003) Economic data. Retrieved February 4, 2016, from http://www.sbp.org.pk/bpd/2003/Anex-CL6.pdf

[CR85] SBP (2015) Economic data. Retrieved March 4, 2016, from http://www.sbp.org.pk/ecodata/fsi/qc/2015/Mar.pdf

[CR86] Schneider W (2008) Merger or acquisition failing? the solution lies in your strategic focus. Retrieved August 4, 2016, from http://ezinearticles.com/?Merger-or-Acquisition-Failing?-The-Solution-Lies-in-Your-Strategic-Focus&id=998205

[CR87] Schuler RS, Jackson SE (2001). HR issues, activities and responsibilities in mergers and acquisitions by. Eur Manag J.

[CR88] Schweiger DM, Ivancevich JM, Power FR (1987). Executive actions for managing human resources before and after acquisition. Acad Manag Exec.

[CR89] Sharma S (2013). Measuring post merger performance—a study of metal industry. Int J Appl Res Stud.

[CR90] Sinkovics RR, Zagelmeyer S, Kusstatscher V (2011). Between merger and syndrome: the intermediary role of emotions in four cross-border M&As. Int Bus Rev.

[CR91] Smith R, Pedace R (2011) The relative importance of IPO and M & A exits for venture capital fund financial performance, pp 1029–1065.

[CR92] Spencer XSY, Joiner TA, Salmon S (2009). Differentiation strategy, performance measurement systems and organizational performance: evidence from Australia. Int J Bus.

[CR93] Surienty L, Ramayah T, Lo MC, Tarmizi AN (2014). Quality of work life and turnover intention: a partial least square (PLS) approach. Soc Indic Res.

[CR94] Syed S (2010) Impact of organizational restructuring on psychological contract breach and attitudes of employees working in private commercial banks of Pakistan. University of Twente. Retrieved from http://essay.utwente.nl/60187/

[CR95] Tetenbaum TJ (1999). Seven key practices that improve the chance for expected integration and synergies. Org Dyn.

[CR96] Van den Heuvel S, Schalk R, Freese C, Timmerman V (2016). What’s in it for me? A managerial perspective on the influence of the psychological contract on attitude towards change. J Organ Change Manag.

[CR97] Wan R (2015) Cultural integration of cross-border M & A activities in the Chinese auto industry. Case study: the acquisition of Geely and Volvo. Retrieved from http://www.theseus.fi/handle/10024/89853

[CR98] Wan HL, Sulaiman M, Omar A (2012). Procedural justice in promotion decisions of managerial staff in Malaysia. Asia Pac Bus Rev.

[CR99] Warter L, Warter I (2015) Can mergers and acquisitions improve banking industry?. Euro and the European banking system: evolutions and challenges. Retrieved from http://s3.amazonaws.com/academia.edu.documents/38017743

[CR100] Weber Y (1996). Corporate cultural fit and performance in mergers and acquisitions. Hum Relat.

[CR102] Weber Y, Tarba SY (2010). Human resource practices and performance of mergers and acquisitions in Israel. Hum Resour Manag Israel.

[CR103] Weber Y, Rachmann-Moore D, Tarba SY (2012). HR practices during post-merger conflict and merger performance. Int J Cross Cult Manag.

[CR104] Wu CM, Chen TJ (2015). Psychological contract fulfillment in the hotel workplace: empowering leadership, knowledge exchange, and service performance. Int J Hosp Manag.

[CR105] Yan S, Zhu Y (2013). Impact of psychological contract violation on interpersonal trust during mergers and acquisitions. Soc Behav Personal.

[CR106] Yang Z, Wang CL (2011). Guanxi as a governance mechanism in business markets: Its characteristics, relevant theories, and future research directions. Ind Mark Manage.

[CR107] Yap BW, Ramayah T, Wan Nushazelin WS (2012). Satisfaction and trust on customer loyalty: a PLS approach. Bus Strategy Ser.

[CR108] Zagelmeyer S, Sinkovics N, Sinkovics RR, Kusstatscher V (2016). Exploring the link between management communication and emotions in mergers and acquisitions. Can J Admin Sci.

[CR109] Zhao X, Lynch JG, Chen Q (2010). Reconsidering Baron and Kenny: myths and truths about mediation analysis. J Consum Res.

[CR110] Zheng N, Wei Y, Zhang Y, Yang J (2016). In search of strategic assets through cross-border merger and acquisitions: evidence from Chinese multinational enterprises in developed economies. Int Bus Rev.

